# Active compression garment prevents tilt‐induced orthostatic tachycardia in humans

**DOI:** 10.14814/phy2.14050

**Published:** 2019-03-29

**Authors:** Kevin L. Kelly, Christopher P. Johnson, Lucy E. Dunne, Brad Holschuh, Michael Joyner, Bruce D. Johnson

**Affiliations:** ^1^ Center for Clinical and Translational Sciences Mayo Clinic Rochester Minnesota; ^2^ Department of Cardiovascular Diseases Mayo Clinic Rochester Minnesota; ^3^ Department of Anesthesia and Perioperative Medicine Mayo Clinic Rochester Minnesota; ^4^ College of Design University of Minnesota Minneapolis Minnesota

**Keywords:** Compression garment, orthostatic intolerance, tilt test

## Abstract

Compression garments are used by patients with lower extremity edema to help regulate fluid distribution and to prevent orthostatic symptoms. Current compression stockings are often reported as uncomfortable and adherence is poor. The current study was conducted to assess the efficacy of a novel active compression garment on healthy individuals undergoing 60‐degree head‐up tilts for 10 min to induce venous pooling and subsequent physiological responses. During tilts while garments were on, participants’ absolute change in heart rate relative to baseline was lower (16.7 ± 8.7 BPM) compared to control (20.9 ± 11.47 BPM,* P* = 0.002). There were no differences in changes in mean arterial blood pressure between conditions (*P* = 0.303). One participant had a pre‐syncopal event which occurred during a tilt without garments. This participant did not experience pre‐syncopal symptoms with the garments on. All together, these data suggest that a novel active compression garment is capable of blunting increases in heart rate during head‐up tilt. While untested thus far in patient populations, these garments may prove efficacious in preventing orthostatic intolerance.

## Introduction

Compression garments are used in patients that have altered fluid distribution and thus require external forces to properly distribute blood and extravascular fluid. Patients complain that these compression stockings are uncomfortable (Anand et al. 2003; Ziaja et al. 2011; Wade et al. 2017) and are particularly difficult to put on and take off (Johnson 2002; Anand et al. 2003), resulting in poor adherence(Anand et al. 2003; Finlayson et al. 2010) and, subsequently, deterioration of health status (Anand et al. 2003).

A novel set of active compression garments, constructed in collaboration with the University of Minnesota College of Design Wearable Technology Lab, was designed to address these limitations. Specifically, they allow for switching on and off the compression such that one may put on and take off the garments with minimal friction. Should the garments become uncomfortable it is easy to deactivate the garments or turn down the magnitude of compression.

Orthostatic intolerance is a condition that is characterized by a severe drop in blood pressure upon changing body posture rapidly, such as standing up from a seated position (Arnold et al. 2018; Lee and Kim 2018). One mechanism that contributes to orthostatic intolerance is a pooling of blood in the lower limbs, where the body struggles to properly return blood to the heart (Arnold et al. 2018). This can be simulated in healthy individuals though a head‐up tilt test, which suspends a person in a vertical position without allowing them to make use of normal mechanisms to augment venous return, such as the muscle pump. This causes a pooling of blood in the venous system of the lower extremities, sequestering 500–600 mL of blood away from the upper body (Dorey et al. 2018). This blood redistribution results in a decrease in blood return to the heart, subsequently causing a decrease in stroke volume, cardiac output, and ultimately blood pressure (Yamanouchi et al. 1998; Dorey et al. 2018). The body will attempt to compensate by increasing heart rate and blood pressure through the baroreceptor reflex. In healthy individuals, the cardiovascular system will be able to do this for an extended period of time before experiencing syncopal symptoms. Individuals with orthostatic intolerance will rapidly experience syncopal symptoms as their body cannot compensate appropriately.

It has been shown that compression garments can be efficacious in counteracting the cardiovascular effects of head‐up tilt, including preventing syncope (Protheroe et al. 2011), increased heart rate (Dorey et al. 2018), decreased cardiac output (Watanuki and Murata 1994; Dorey et al. 2018), and blood pressure (Denq et al. 1997). Previous studies have shown that blood pressure responses to compression garments during tilt or to prevent orthostatic hypotension are mixed (Smeenk et al. 2014; Dorey et al. 2018).

The aim of this study was to determine if a set of novel active compression garments were sufficient to mitigate the physiological consequences of head‐up tilt in healthy humans during a 10‐min, 60‐degree tilt test.

## Methods

This study was reviewed and approved the Mayo Clinic Institutional Review Board and complies with the Declaration of Helsinki. All participants provided written informed consent prior to participation in this study. The results presented in this submission were completed under IRB# 16‐005735, approved on August 5, 2016.

All participants were healthy adults free of cardiovascular disease and free of medication affecting cardiovascular health. All participants were nonsmokers, and were asked to abstain from alcohol and exercise for 24 h, and food and caffeine day‐of. Ages ranged from 21 to 37 years, with a BMI of range of 20.3–27.4 (Table 1). All participants were screened for leg size, necessary as there was only one set of garments with a specific size restriction. Measurements made include ankle, calf, knee, thigh and groin circumference, and the distances between each. Average lower leg and thigh lengths were 34.2 ± 1.6 cm and 31.6 ± 1.5 cm, respectively.

**Table 1:** Demographics. All values reported as mean (SD)

	Overall (*N* = 10)
Sex	
Female	6
Male	4
Age (years)	28.2 (4.3)
Height (cm)	168.2 (8.5)
Weight (kg)	68.2 (9.6)
BMI (kg/m^2^)	24.0 (2.4)
Lower leg length (cm)	34.2 (1.6)
Upper leg length (cm)	31.6 (1.5)

Participants were re‐measured the morning of the tilt test to ensure no significant changes in leg size. Participants were asked to lay supine while investigators properly affixed the garments to the participants’ legs and thighs. Participants were instrumented with a standard 3‐lead ECG and finger plethysmograph for heart rate and beat to beat blood pressure, respectively. After 5‐min quiet supine rest, the tilt table was tilted to 60‐degrees for 10 min and then returned to supine for 5‐min recovery. This 20‐min protocol was conducted twice with each participant, once with the garments and once without. The order was randomized for each participant.

The garments, designed in collaboration with the Wearable Technology Lab at the University Of Minnesota College Of Design, are previously described. A detailed investigation of the active garment system design and performance has been conducted (Duvall et al. 2017; Granberry et al. 2017; Pettys‐Baker et al. 2018; Schleif et al. 2018). In short, four separate garments were created to be affixed to the legs, one on each calf and thigh. Each garment consisted of two layers. The base layer in contact with the participant's skin was made of a Teflon material and was designed to separate the participant from the second layer, which consisted of a series of coils that are fairly pliable at room temperature. Each thigh garment had three bands and each calf garment had seven bands of coils spaced evenly along the length of the leg. When heated, these coils constrict together to produce a compression effect. To produce heat, a current is passed through the coils.

All statistics were performed in JMP 10.0. All values are reported as mean ± standard deviation.

## Results

**Figure 1:**
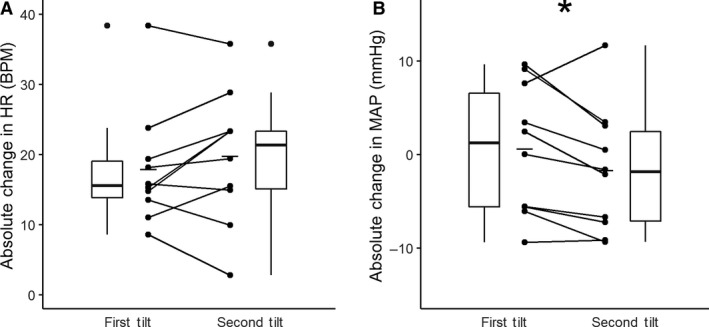
Tilt order. There were no statistical differences in the absolute change in heart rate (HR) (*P* = 0.267, paired Students *t*‐test). Absolute change in mean arterial pressure (MAP) from baseline decreased and became negative during the second tilt compared to the first tilt (*P* = 0.041, paired Students *t*‐test). Connected points represent a single participant's responses. Mean response denoted by solid line. Box plots show median, maximum, minimum, first and third quartiles, and outliers.

**Figure 2:**
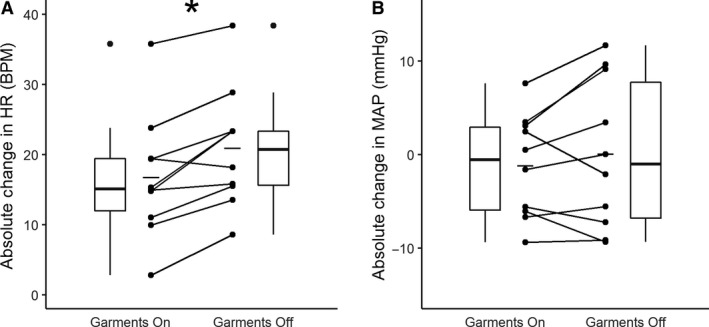
Garment condition. Absolute change in heart rate (HR) from baseline to tilt was significantly lower during the trial with garments on compared to the trial with garments off (*P* = 0.002, paired Student's *t*‐test). There were no statistical difference in absolute change of mean arterial pressure (MAP) change from baseline to tilt between conditions (*P* = 0.303, paired Student's *t*‐test).

During the tilt phase of the protocol, participants’ heart rate increase relative to baseline was not significantly different between the first tilt (17.8 ± 8.4 BPM) and the second tilt (19.7 ± 9.4 BPM), independent of condition order (Fig. 1A; *P* = 0.267, paired Student's *t*‐test). During the tilt phase of the protocol, participants’ absolute change in mean arterial blood pressure was lower during the second tilt (−1.7 ± 6.7 mmHg) compared to the first tilt (0.6 ± 7.0 mmHg, Fig. 1B, *P* = 0.041, paired Student's *t*‐test). Mean arterial pressures during tilt were not significantly different from their respective baselines in both the first (82.9 ± 10.0 vs. 83.4 ± 5.7 mmHg, *P* = 0.803, paired Student's *t*‐test) and second tilt (85.3 ± 11.7 vs. 83.6 ± 9.5 mmHg, *P* = 0.432, paired Student's *t*‐test).During the tilt phase of the protocol, heart rate increased by 16.7 ± 8.7 BPM relative to baseline while the garments were on, compared to 20.9 ± 11.47 BPM during control (Fig. 2A; *P* = 0.002, paired Student's *t*‐test), and decrease in mean arterial blood pressure from baseline was not significantly different between the garment (−1.2 ± 5.5 mmHg) and control (0.0 ± 8.0 mmHg) conditions (Fig. 2B, *P* = 0.303, paired Student's *t*‐test).

**Figure 3:**
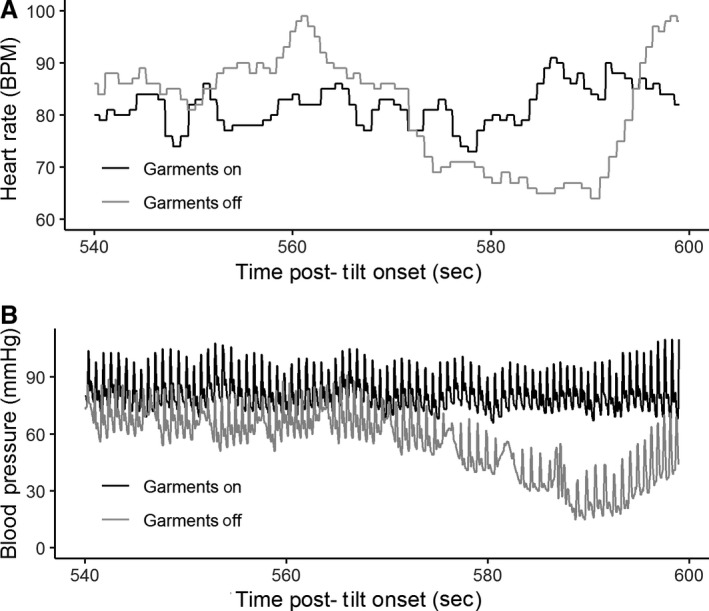
Pre‐syncope. One participant experienced syncope during the study. Syncopal symptoms can be seen in the garments off condition starting at approximately 570 sec after the tilt phase began.

Of all participants and tilts, there was only one instance of pre‐syncope that was quickly resolved by returning to a supine position. This event occurred during the second tilt after approximately 9 min and 50 sec. The participant was not wearing compression garments during this event. A visualization of this event and the corresponding time points of the first tilt are shown in Figure 3.

**Figure 4:**
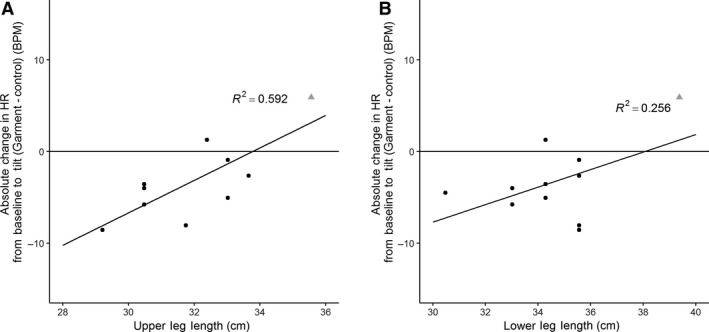
Leg length and response to tilt. There is a correlation between upper leg length and the difference in absolute change in heart rate between garment on and garment off conditions (*r* ^2^ = 0.592, Pearson correlation). There is no correlation with lower leg length and difference in absolute change in heart rate between garment on and garment off conditions (*r* ^2^ = 0.256). An 11th participant was included in this figure and analysis to demonstrate importance of garment fit (see grayed triangle in figure). This 11th participant was not included in any other figures or analysis.

Upper leg sizes were correlated with the absolute change in heart rate from baseline to tilt during the garment condition (Fig. 4A, *r* ^2^ = 0.592, Pearson correlation). Lower leg lengths were not correlated with the change in heart rate from baseline to tilt during the garments condition (Fig. 4B, *r* ^2^ = 0.256, Pearson correlation).

One participant's legs were longer than the maximum length that the garments were designed for. This participant's data are included in Figure 4A and B to illustrate the importance of leg size, but excluded from other figures and analysis.

There were no significant differences based on sex.

## Discussion

These data show that these novel active compression garments are capable of producing physiological changes during a tilt test consistent with a decrease in venous pooling.

There was no significant difference in absolute change in heart rate to tilt between the first and second trials, regardless of condition order (Fig. 1A). Comparing the first and second tilts, mean arterial pressure (MAP) was decreased relative to baseline during the second tilt (Fig. 1B), though MAP was not significantly different from respective baseline during either tilt. While participants were randomized to condition order, there was concern that repeated tilt tests would result in an exaggerated response during the second tilt.

Absolute change in heart rate relative to baseline was significantly lower during tilt when garments were worn compared to when they were not (Fig. 2A). This suggests that the compression garments induce a decrease in venous pooling, which facilitates an increase in venous return to the heart, resulting in a higher stroke volume and therefore reducing heart rate. Absolute change in mean arterial blood pressure relative to baseline was not different between conditions (Fig. 2B). This change in heart rate and lack of change in mean arterial blood pressure has been previously reported by standard non‐active compression garments (Dorey et al. 2018). This suggests that these active garments act similarly as normal compression garments.

Of 11 participants, there was only one instance of pre‐syncopal symptoms (Fig. 3). This event occurred at approximately minute 9:50 of the second tilt, during which they did not have the garments on. This participant did not experience any symptoms during the tilt in which the garments were on. This supports previous findings that compression garments are capable of preventing syncope (Protheroe et al. 2011).

The relationship between leg size and difference in change from baseline between conditions (Fig. 4A) supports previous findings that the fit of compression garments is important in producing the intended cardiovascular changes (Protheroe et al. 2011).

Taken together, these data suggest that these garments can serve to improve venous return to the heart. While still untested in clinical populations, this concept may be useful for patients with orthostatic intolerance who would normally be prescribed to wear compression stockings. These novel active compression garments have several advantages over traditional compression stockings. First, because these garments activate to cause compression, they are not tight when donning and doffing them, which with further concept development may prove beneficial. Second, patients can turn off the compression if it becomes uncomfortable instead of the complete removal of the garments. This might make patients more likely to adhere to their compression regimen and improve patient outcomes.

## Conflict of Interest

The authors of this paper have no conflicts of interest to disclose.
